# Supplementary figures and images for: Enhancement of Macrophage Immunity against Chlamydial Infection by Natural Killer T Cells
Source: Cells. 2024 Jan 11;13(2):133. doi: 10.3390/cells13020133 (PMC10813948; doi:10.3390/cells13020133)

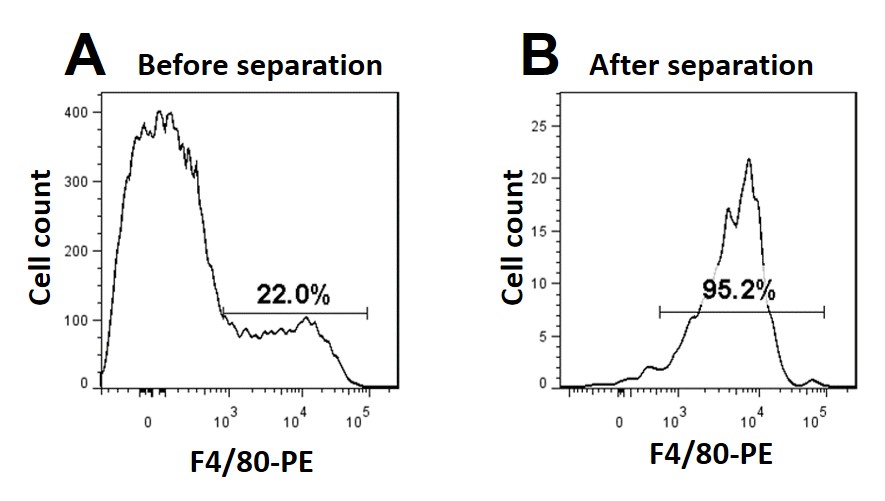

Supplement: Supplementary file 1 [file cells-13-00133-s001.zip › cells-2747034-supplementary.jpg]
